# A Core-Offset Mach Zehnder Interferometer Based on A Non-Zero Dispersion-Shifted Fiber and Its Torsion Sensing Application

**DOI:** 10.3390/s16060856

**Published:** 2016-06-10

**Authors:** Eduardo Huerta-Mascotte, Juan M. Sierra-Hernandez, Ruth I. Mata-Chavez, Daniel Jauregui-Vazquez, Arturo Castillo-Guzman, Julian M. Estudillo-Ayala, Ana D. Guzman-Chavez, Roberto Rojas-Laguna

**Affiliations:** 1 Departamento de Estudios Multidisciplinarios, División de Ingenierías, Universidad de Guanajuato, Av. Universidad s/n, Col. Yacatitas, Yuriría Gto. C.P. 38940, Mexico; eduardoh9@hotmail.com (E.H.-M.); ruth@ugto.mx (R.I.M.-C.); ad.guzman@ugto.mx (A.D.G.-C.); 2 Departamento de Ingeniería Electrónica, División de Ingenierías, Universidad de Guanajuato, Carretera Salamanca-Valle de Santiago km 3.5 + 1.8, Comunidad de Palo Blanco, Salamanca Gto. C.P. 36885, Mexico; jaureguid@ugto.mx (D.J.-V.); julian@ugto.mx (J.M.E.-A.); rlaguna@ugto.mx (R.R.-L.); 3 Facultad de Ciencias Físico-Matemáticas, Universidad Autónoma de Nuevo León, Av. Universidad s/n, Cd. Universitaria, San Nicolas de los Garza Nuevo León C.P. 66455, Mexico; arturo.castillogz@uanl.edu.mx

**Keywords:** Mach-Zehnder interferometer, fiber optics, fiber optic sensors, non-zero dispersion-shifted fiber

## Abstract

In this paper, an all-fiber Mach-Zehnder interferometer (MZI) based on a non-zero dispersion-shifted fiber (NZ-DSF) is presented. The MZI was implemented by core-offset fusion splicing one section of a NZ-DSF fiber between two pieces of single mode fibers (SMFs). Here, the NZ-DSF core and cladding were used as the arms of the MZI, while the core-offset sections acted as optical fiber couplers. Thus, a MZI interference spectrum with a fringe contrast (FC) of about 20 dB was observed. Moreover, its response spectrum was experimentally characterized to the torsion parameter and a sensitivity of 0.070 nm/° was achieved. Finally, these MZIs can be implemented in a compact size and low cost.

## 1. Introduction

Different types of optical setups have been proposed for sensing physical and chemical parameters such as pressure, temperature, curvature, refractive index and torsion [1,2,3,4,5]. Many of these are based on the Mach-Zehnder interferometer (MZI) [6,7,8,9,10,11,12,13,14,15,16,17,18,19], because, it has a simple optical configuration, low cost, high sensitivity and compact size. For this reason, several MZIs have been implemented by using different fabrication techniques such as; optical fiber tapers [6,7], thin core fibers (TCF) [8], long period grating cascade structures [9,10], Photonic crystal fiber (PCF) [11,12,13] and by splicing optical fibers with a core offset section [14,15,16,17,18,19,20,21,22]. Hence, there can be found in literature a large number of sensing arrangement designs based on a MZI. For instance, a MZI based on a core offset attenuator was proposed by [14], in which a displacement sensor with a sensitivity of −0.66 dB/µm and a maximum extinction ratio over 10 dB was achieved. Other examples of MZIs based on the core-offset technique can be found in works proposed in [15,16,17], where the authors reported refractive index sensors with sensitivities of 0.333, 28.2 and 78.7 nm/RIU, respectively. Further examples of a MZI based on core-offset sections were proposed in [18,22], here the curvature sensors with sensitivities of −0.88 dB/m, −22.99 nm/m were respectively achieved. Yao *et al.* [19] proposed a MZI based on the core-offset technique with a fiber Bragg grating, in which a simultaneous measurement of refractive index and temperature was achieved. The experimental results showed sensitivities of 13.75 nm/RIU and 0.04 nm/°C respectively. Basically, all these MZIs based on the core-offset technique have been implemented for sensing some known physical parameters (displacement, curvature, refractive index and temperature), but they have not been implemented for sensing the physical effect of torsion. Recently, some torsion sensing setups based on intermodal interferometers have been presented [3,23,24,25]. For instance, Kim *et al.* [3] performed a torsion sensing based on a Sagnac interferometer and the sensitivity was about of 0.06 nm/°. A further example is a torsion sensing setup based on a three beam optical path MZI, with a sensitivity of 0.01 nm/°, which was reported in [23]. In addition, a torsion sensor has also been reported in [24]; the sensor was based on a helical waveguide, and depicted a torsion sensitivity of 0.04 nm/°.

In this work, an all MZI based on a non-zero dispersion-shifted fiber (NZ-DSF) was experimentally demonstrated. We design our MZI by core offset fusion splicing of a segment of NZ-DSF between two SMFs. Moreover, the principal operation is discussed, as well as some experiments results and its spectral response characterization, is also provided.

## 2. MZI Fabrication Process and Operation Principle

### 2.1. The Core-Offset Fusion Splicing of a NZ-DSF and SMF

**Figure 1:**
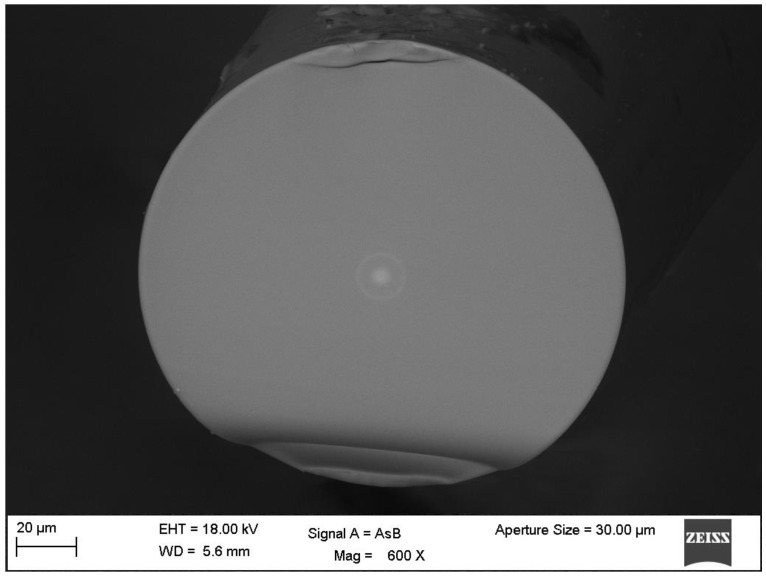
Image of Non-zero dispersion-shifted fiber cross section.

For the purpose of fabricating the MZI, two conventional single mode fibers (SMF-28) and a segment of a non-zero dispersion-shifted fiber (Corning Fiber Model SMFLF) were used. In Figure 1 a cross-section microscope image of the used NZ-DSF is shown. Here, it can be observed that the NZ-DSF has a core diameter of 5.8 µm, a cladding diameter of 125 µm and a ring core diameter of 16 µm but it is important to point out that, this ring core has only a thickness of 3 µm. Moreover, the effective refractive indexes layers are: $n_{\text{core}}=1.4598,$ $n_{\text{ring}}=1.4498,$ $n_{\text{clad}}=1.4458$ for the core, ring core and cladding respectively [10].

**Figure 2:**
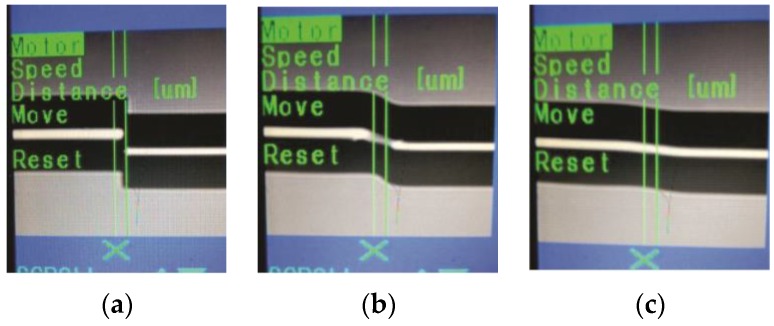
Fabrication process of the Mach-Zehnder interferometer (MZI) (**a**) Initial position with a core offset section with lateral of 30 µm; (**b**) Splice joint between single mode fiber (SMF)|non-zero dispersion-shifted fiber (NZ-DSF); (**c**) Spliced image of SMF|NZ-DSF.

**Figure 3:**
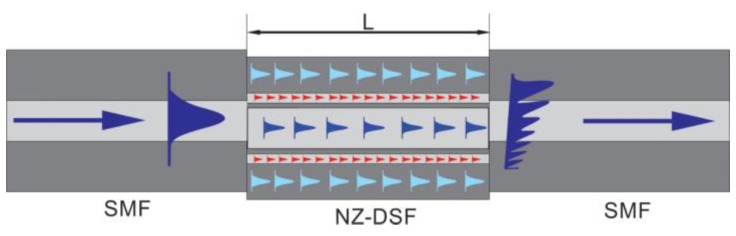
The Schematic diagram of Mach-Zehnder Interferometer.

In this way, in order to implement the MZI, both optical fibers were fixed into the splicer machine fiber holders (Fitel S175) and by manual mode, the NZ-DSF was displaced downward for a distance of 30 µm (See, Figure 2a). It is important to mention that this distance has been chosen after performing several experimental characterizations with the lateral shifting of the core sections of both optical fibers and as the maximum fringe contrast values were achieved. This is due to the recoupling increase between the core, ring core and cladding modes with the increment of the lateral displacement of the core offset sections [16]. Afterwards, 20 discharges were applied over the joint SMF-NZ-DSF, and as a result, the optical fibers were spliced, as it is shown in Figure 2b. The next step was to splice a determined length of NZ-DSF and the SMF (See, Figure 2c), which, it was carried out following the same method for the first splice and as a result, a SMF|NZ-DSF|SMF structure was achieved. Hence, the core and cladding are considered as the arms of the MZI while the splicing joints act as optical fiber couplers, as it can be observed in Figure 3. Finally, in order to obtain the splicing joints, the splicer machine was programed with the following parameters: (a) 91 of arc power; (b) 240 ms of prefusion time and (c) 750 ms of arc duration.

### 2.2. Priciple of Operation

The principle of operation of this MZI can be explained as follows: when the fundamental mode travels through the SMF until it reaches the first core-offset joint (See, Figure 3), it is refracted into the NZ-DSF. As a result, core and cladding modes are excited. Due to the effective refractive index difference among the core, ring core and cladding modes, a phase difference can be produced through the same physical length. Hence, as this fiber has a core, ring core and cladding structure, in practice, each one will behave as an optical path [23].

Thus, the phase difference for this type of MZI, among the core mode, ring core modes and cladding modes, can be expressed by $∆\phi =2\pi ∆n_{e}L/\lambda$ [16], where $∆n_{e}$ is the NZ-DSF core, ring core and cladding effective refractive index differences. λ represents the operating wavelength and L is the length of NZ-DSF segment. Then, the core mode, ring core modes and cladding modes travel throughout the NZ-DSF until the second core offset region of the SMF|NZ-DSF|SMF structure, where the ring core modes and cladding modes are re-coupled to the core mode. Thus, the core-offset joints between the SMF and NZ-DSF act as optical couplers, while the core, ring core and cladding sections of the NZ-DSF act as the MZI arms [26]. Hence, it is possible to consider this MZI as an intermodal interferometer, in which the spectral fringe separation is given by [27].

(1) $$∆\lambda =\frac{\lambda^{2}}{{∆n}_{e}L}$$

On the other hand, since the phase difference and separation fringes are wavelength dependent, the transmitted optical power, by the interferometer, will be present as a maximum at certain wavelength and a minimum at another [27]. It is important to point out that, the maximum and minimum values are used to obtain the fringe contrast of the transmitted optical power. In this way, this fringe contrast is a very important parameter for physical sensing applications, since with a higher fringe contrast a more accurate measurement can be achieved [28].

## 3. Characterization of MZIs with a NZ-DSF

### 3.1. Experimental Setup

**Figure 4:**
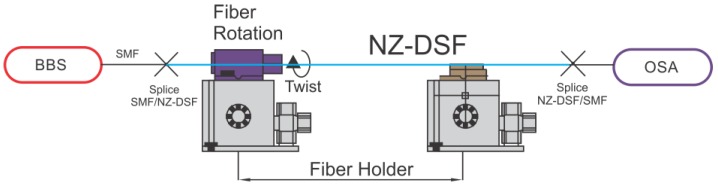
Experimental setup to characterized MZI.

In order to characterize the MZI optical spectrum response, the experimental setup shown in Figure 4 was used. Here, the light of a pumping diode (Qphotonics, model QFBGLD-908-150J, Ann Arbor, MI, USA) was coupled to a 2.8 m erbium doped fiber (Thorlabs, model M5-980-125, Newton, NJ, USA) to generate a broadband source (BBS), from 1450 to 1650 nm. This light was launched into the MZI structure. The output spectrum was recorded by an optical spectrum analyzer (OSA, Yokogawa AQ6370C, Tokyo, Japan) with a resolution of 0.02 nm.

### 3.2. Characterization of the MZI with Different Lengths of NZ-DSF

**Figure 5:**
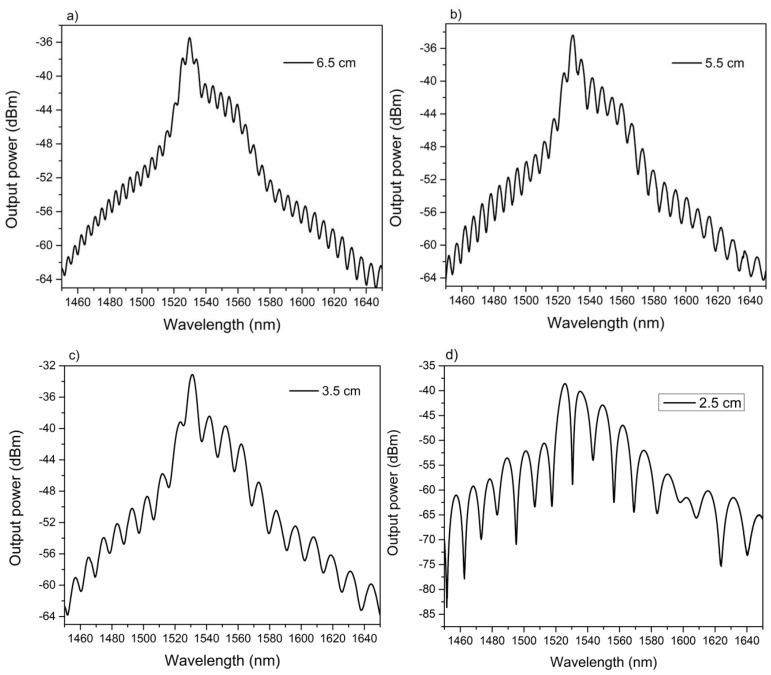
Output spectra of the MZIs with different lengths of NZ-DSF (**a**) *L* = 6.5 cm; (**b**) *L* = 5.5 cm; (**c**) *L* = 3.5 cm; (**d**) *L* = 2.5 cm.

In this way, the output spectra of four MZIs with different physical length (*L*) were investigated. It is important to mention that the lateral offset of all these MZIs was 30 µm. The physical lengths of the NZ-DSF were *L* = 6.5, 5.5, 3.5 and 2.5 cm respectively. The output spectra using the lengths of 6.5 and 5.5 cm are shown in Figure 5a–c respectively. Here, it can be observed that the separation between two consecutive spectral fringes (Δλ) was 3.6, 4.92, 6.20 and these values almost remained constant during all wavelength spectrum. As a result of these constant values, a periodic sinusoidal curve was achieved [29]. Hence, we can highlight that these three output spectra have the behavior of a two-mode MZI. On the other hand, Figure 5d depicts the output spectrum of a MZI with a length of 2.5 cm where a Δλ of 14.38 nm was achieved. However, some little variations of the Δλ values were obtained at different wavelength regions of the output spectrum. And consequently, an aperiodic sinusoidal curve was obtained [30]. This can be explained as follows: since the NZ-DSF contains three different effective refractive indexes and the device is fabricated in a short length, then the ring core mode contributes as another beam optical path. So, in the second core-offset region three kinds of beam interference can be produced [31,32]. The first one, will be due to the interference between the core and the ring core modes and the second one will be carried out between the core and cladding modes [23]. At this point, it is important to mention that due to the core-offset, the excited modes in the ring core region (~3 µm) are quite comparable with the ones in the cladding region (125 µm) because of its size. For this reason, we believe that the contribution of the ring modes is not significant for larger lengths but it is highly important for shorter lengths. Besides, the fringes contrast was also modified with respect to the different lengths, and their observed values were 2.25, 4.27, 5.22 and 20.54 dB in a wavelength region from 1540 to 1541 nm respectively. The fringe contrast of 20 dB for a core-offset MZI of *L* = 2.5 cm, is higher than for other devices FC, implemented by using different techniques. For instance, it is around two times higher than at a MZI based on two cascaded long-period gratings (12 dB) [33], and a MZI based on in-series fattened fiber gratings (10 dB) [9]. Meanwhile, this contrast is also higher than in a MZI based on concatenating a single mode abrupt taper and core-offset section (10 dB) and for a core-offset MZI implemented by using SMF (9 dB) [15,17], or a core-offset MZI based on a polarization maintaining fiber. Finally, the fringe contrast is similar to the reported in [12], where the author used a photonic crystal fiber (18 dB). Thus, as mentioned above, a higher fringe contrast value is important due to the fact that it will induce a more accurate physical measurement. Besides, the core-offset MZI fabrication process is chipper and simpler than for the LPG, PCF and tapered fiber process. Also, it can be observed that in the length of 2.5 cm (See, Figure 5d), the output spectrum has an irregular shape and may be caused by the superposition of many cosine curves, which corresponds to the interference between the core and high order cladding modes, respectively [16].

**Figure 6:**
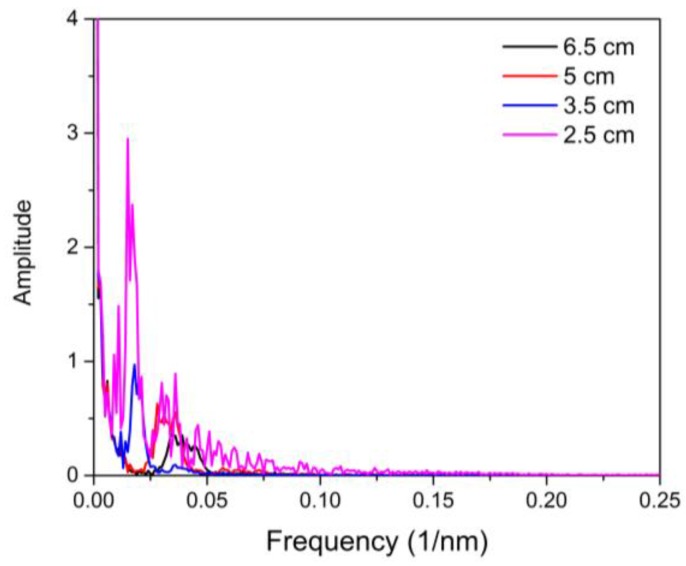
Frequency spectra of the MZIs with NZ-DFS lengths.

In order to determine the cladding modes and ring core modes that construct the interference spectrum, the fast Fourier transform (FFT) of the wavelength spectra is performed as is shown in Figure 6. It is found that the spatial frequency of the MZIs with lengths of L = 6.5, 5.5 and 3.5 cm, have only one dominant peak amplitude and they are located at 0.04104 0.03221 and 0.0175 1/nm. Furthermore, for the interferometer with length of 2.5 cm, the spatial frequency shows two values of spatial frequency localized at 0.0144 and 0.03221 1/nm (See, Figure 6). Thus, we believe these two frequencies are the dominant cladding mode and dominant ring core mode. Hence, the spectrum patterns will be produced by the coupling between the fundamental mode, ring core mode and high order cladding mode. However, it can be appreciated that the amplitude of the frequency localized at 0.03221 1/nm, is much smaller than the other frequency. In this way, as mentioned above the contribution of the ring core mode is small when the fringe interference is produced. The insertion losses varied among devices with a typical value about of 4 dB.

## 4. Experimental Results and Discussions

**Figure 7:**
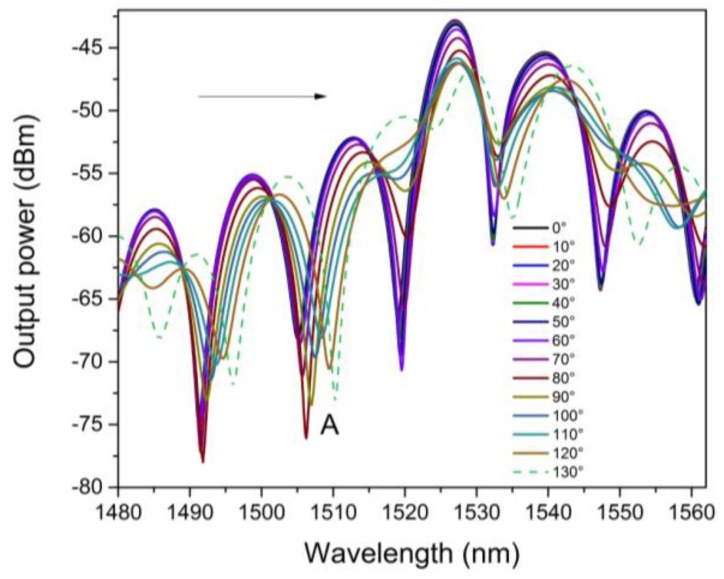
The Wavelength shifting as torsion function.

**Figure 8:**
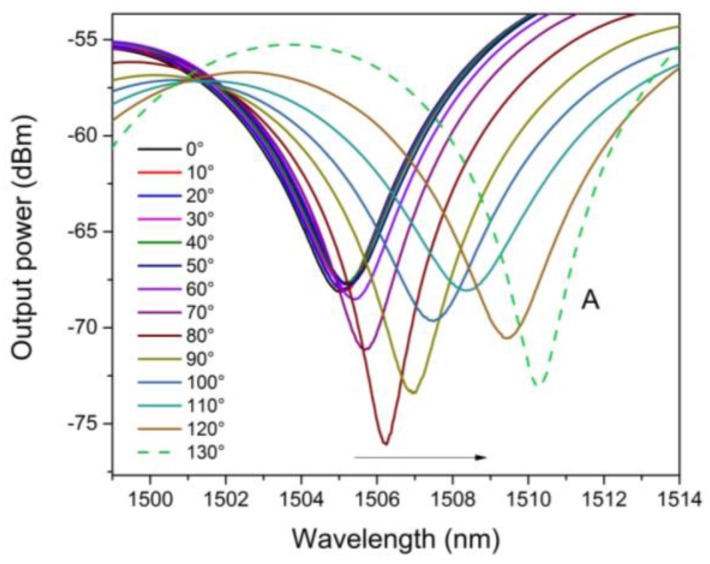
The Interference spectrum to dip centered at 1504 nm.

A MZI with length of 2.5 cm was tested as a torsion sensor. Here, the MZI was mounted over a fiber rotator stage (Thorlabs, model HFR007) to apply the torsion effect (See, Figure 4). In this way, the MZI was twisted in a clockwise direction from 0° to 130° with steps of 10°. In Figure 7, the spectral shifting of the MZI, can be observed, under different torsion angles. Moreover, in order to determine the spectral changing, measurement of a called *A* dip was done which is centered wavelength at 1504 nm (See, Figure 8). Here, it can be appreciated that the wavelength of this dip was shifted from 1504 to 1511 nm, corresponding to a total wavelength shift of about 10 nm. Hence, this spectral shift is produced due to the photoelastic effect induced by the torsion applied to the MZI [33].

Moreover, a shear stress along the circumferential direction occurred when the torsion is applied. As a result of the stress, the effective refractive indexes of the core, ring core and the cladding are changed [23]. Since, the NZ-DSF has a cladding diameter (125 µm), that is, much larger that the core (5 µm), the shear stress is almost zero for the core and ring core thickness compared with the cladding. Hence, the maximum shearing stress will occur at the most remote points from the core center [33,34].

**Figure 9:**
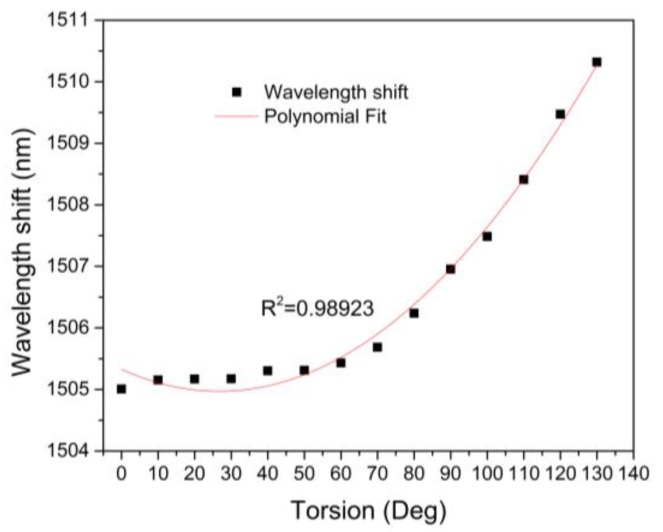
The Interference spectrum to dip centered at 1504 nm.

Therefore, the change of the effective refractive index both for the core and ring core thickness are small, which means a growth on the effective refractive index of cladding. In this way, modifying the torsion effect can change the effective refractive index difference between cladding and ring core thickness and core $\left(∆n_{e}\right)$. In Figure 9, a dip (at 1504 nm) in behavior is seen, which shows a nonlinear relationship between the wavelength spectral shifting and the torsion applied. Because of core-offset fusion splicing, the shear stress would not be constant throughout the SMF|NZ-DSF|SMF structure, since, it depends on the length, the radius and torsion rate [30]. Moreover, as the core offset sections change abruptly the diameter through of all the MZI structure, large perturbations of shearing stress take place [35]. In this way, a nonlinear shearing stress is obtained due the difference between the two diameters (core-offset sections) [35]. As a result, the torsion effect is not uniform along the circumferential direction of the MZI (See, Figure 9). Hence, the maximum sensitivity of our sensor was observed at a torsion range from 60° to 130°. Besides, in this torsion range, a quasilinear relationship can be seen between the spectral fringe and the torsion applied and also that a maximum wavelength shifting was achieved. In this way a sensitivity value of 0.070 nm/° and a $R^{2}$ values of 0.989 were reached. Here, it is important to point out that, the maximum sensitivity is almost always present in the fiber optic sensors in the higher values [28]. At this point, it is important to mention that room temperature remained constant, in order to carry out all the experimental characterizations. Finally, the MZI was kept in a straight line to avoid polarization effects during the experiment.

## 5. Conclusions

We have proposed and demonstrated a new Mach-Zehnder interferometer based on a non-zero dispersion shifted fiber. Here, the MZI was implemented by a core offset splicing of a NZ-DSF between two segments of SMF, and several fringe contrast of 2.25, 4.27, 5.22 and 20.54 dB were obtained respectively. Moreover, the MZI was tested for torsion sensing and it was observed that the torsion effect induced a nonlinear behavior due to the shearing stress that is not uniform at the core-offset MZI structure. Here, by experimental measurements, we determined a sensitivity of 0.070 nm/° to a physical length of the NZ-DSF of 2.5 cm.
